# Neurocognitive Monitoring and Care During Pediatric Cardiopulmonary Bypass—Current and Future Directions

**DOI:** 10.2174/157340308784245766

**Published:** 2008-05

**Authors:** Jennifer K Lee, R Blaine Easley, Kenneth M Brady

**Affiliations:** Departments of Anesthesiology/Critical Care Medicine and Pediatrics, Johns Hopkins Medical Institutions, Baltimore, MD, USA

## Abstract

Neurologic injury in patients with congenital heart disease remains an important source of morbidity and mortality. Advances in surgical repair and perioperative management have resulted in longer life expectancies for these patients. Current practice and research must focus on identifying treatable risk factors for neurocognitive dysfunction, advancing methods for perioperative neuromonitoring, and refining treatment and care of the congenital heart patient with potential neurologic injury. Techniques for neuromonitoring and future directions will be discussed.

## INTRODUCTION

Advances in pediatric cardiac surgery have significantly improved morbidity and mortality for children with congenital heart disease (CHD), bringing new hope to patients and their families, but children who survive the perioperative period still face developmental challenges as they grow older. The developing brain is vulnerable to insults that can have lifelong effects. The pediatric brain’s rapid neuronal proliferation and development, heavy axonal growth, fragile vasculature, and high metabolic rate place it at high risk for ischemia and reperfusion injury [1,2]. These insults can occur preoperatively, during surgery, and postoperatively. Attention is now shifting toward minimizing perioperative neurologic injury to prevent long-term consequences.

Too often, the neurologic insults sustained during or soon after surgery are not recognized for years, until the children manifest behavior and learning problems in school. Children with surgically corrected CHD are more likely to perform poorly on neurodevelopmental tests and require remedial educational services compared to healthy children [3-6]. In addition to providing life-sustaining cardiac care in the perioperative period, standardizing care for these children to maximize their collective functional futures is the imperative of our specialty.

## NEUROLOGIC INJURY AND CARDIAC SURGERY

### Preoperative Risk Factors

Multiple factors increase the risk of neurocognitive impairment in pediatric heart disease (Table **1a**). Children with CHD have a higher incidence of congenital malformations of the central nervous system (CNS) [1,7-9]. Abnormal in utero CNS development may be associated with a genetic syndrome or may occur as an independent disease entity. Acquired brain injuries may be secondary to the CHD or to a complicated preoperative course. McQuillen *et al.* prospectively examined the MRIs of 62 newborns with CHD preoperatively [10]. Evidence of brain injury was present in 37% of the newborns and was more common with two-ventricle heart defects. The need for balloon atrial septostomy, primarily for transposition of great arteries (TGA), was a significant and independent risk factor for focal stroke and other preoperative brain injuries.

### Cardiac Surgery

Successful surgical correction of a structural heart lesion improves a child’s neurodevelopmental potential by preventing long-term hypoxia and hemodynamic instability. However, the stress and inflammatory response associated with the surgery itself contributes to cerebral insult [11]. Many pediatric cardiac surgeries also require cardiopulmonary bypass (CPB), which greatly increases the risk of brain injury.

### Cardiopulmonary Bypass

CPB carries significant risks to the brain. (Table **1b**) The basal ganglia are particularly vulnerable to ischemia during CPB [12,13]. Metabolite indicators of neuronal injury, such as increased glutamate, decreased N-acetylaspartate, and increased myoinositol, are reported following bypass in children [14].

Maintaining cerebral autoregulation during the bypass period is crucial in preventing brain injury during cardiac surgery. Through cerebral autoregulation, the healthy brain maintains constant cerebral blood flow (CBF) across a wide range of cerebral perfusion pressures (CPPs) to match the brain’s oxygen needs with supply. Mechanisms for cerebral autoregulation function within a specific range of arterial pressures. When mean arterial pressure (MAP) drops below or exceeds the boundaries of this range, CBF becomes passive to systemic blood pressure. CBF that is pressure passive due to low MAP results in cerebral ischemia, and pressure passivity due to high MAP causes hyperperfusion injury. The lower limit of autoregulation (LLA) in adults was initially described by the Lassen curve, and placed at a CPP of 50 mmHg [15]. These data were derived from pharmacologic manipulation of pregnant volunteers, and the true LLA in adults is less clear than once thought [16].

Pediatric-specific autoregulatory ranges are even less clearly defined. In a limited study of infants and children (13 subjects <2 years of age) who underwent anesthesia, no age-related differences were seen in the LLA, but the lower limit reserve (MAP – LLA) was significantly lower in younger patients [17]. Autoregulatory limits may vary according to gestational and chronologic age, the type of structural heart disease, and the timing of surgical repair, but these limits have not been defined beyond theoretical consideration. For instance, does an infant with an aortic coarctation have a different cerebral autoregulatory range from other infants of the same age, analogous to the observation in adults with chronic hypertension?

Adult studies have shown that CPB impairs cerebral autoregulation. Some anesthetics, such as isoflurane, further disrupt autoregulation during bypass [18]. When autoregulation is impaired, the range of tolerable CPPs at which the brain can appropriately match CBF to metabolic needs becomes narrow in comparison to a healthy brain, consequently narrowing the hemodynamic targets of care during and after CPB.

Without knowing a patient’s autoregulatory blood pressure boundaries, it is extremely difficult to keep hemodynamic parameters within the ideal range, where the brain can match CBF to changing oxygen needs. Inappropriate bypass flow rates, excessive or inadequate anesthesia, vasopressors, and vasodilators can lower or raise MAP beyond the autoregulatory thresholds, placing the brain at significant risk of ischemia or hyperperfusion injury with low or high MAPs, respectively.

Several reports have been published of impaired cerebrovascular autoregulation in children with CHD, although it is unclear if these children had altered autoregulation preoperatively or if impairment occurred secondary to surgery and CPB. Hayashida *et al.* reported that children younger than 4 years were more likely to suffer cerebral ischemia during cardiac surgery than were older children [19]. The authors theorized that these patients had immature cerebral autoregulation, which made them more vulnerable to brain ischemia. Hoffman *et al.* found that children with hypoplastic left heart syndrome (HLHS) after stage 1 palliation had persistently elevated cerebral vascular resistance with compromised cerebral oxygenation, despite increasing brain oxygen requirements post-bypass [20].

Hypotension during weaning from bypass and in the immediate post-bypass period is particularly deleterious. Cerebral metabolism and oxygen consumption resume during rewarming and reperfusion. However, increased cerebrovascular resistance and altered tissue microcirculation post-bypass decrease oxygen delivery to the brain [20-22]. These factors, combined with impaired cerebral pressure autoregulation from bypass, increase the risk of brain ischemia, particularly during hypotension.

Obstructed cerebral venous drainage may also affect CBF by decreasing CPP. Theoretically, high pressure in a cavopulmonary anastomosis from pulmonary hypertension or anastomosis obstruction can impede cerebral venous drainage and decrease CBF.

### Postoperative Factors

The brain remains at substantial risk of ischemia after cardiac surgery (Table **1c**). Impaired cerebral autoregulation is particularly detrimental, as rising cerebral oxygen needs postoperatively may not be satisfied. Hoffman *et al.* reported that cerebral autoregulation in neonates remained disrupted for at least 24 hr following hypothermic CPB [23]. In newborns following repair of CHD, low MAP on the first postoperative day was significantly associated with MRI evidence of newly acquired, postoperative white matter injury [10].

The cerebrovascular response to hypoxemia is also altered following bypass. Brain metabolism and oxygen demand above baseline can persist for more than 24 hr after CPB [24]. In piglets after deep hypothermic circulatory arrest (DHCA), CBF no longer increased in response to hypoxemia despite normal MAPs and normal CPPs [25].

Patients with bidirectional cavopulmonary anastomoses rely upon venous pressure from upper body venous drainage to drive pulmonary blood flow. In neonates, the venous pressure that determines this upstream gradient largely comes from cerebral drainage. A pediatric study of bidirectional cavopulmonary anastomoses in the postoperative period demonstrated that mild hypercapnia with PaCO_2_ of 45–55 mmHg induced cerebral vasodilation, improved pulmonary blood flow, and increased oxygen supply [26]. However, the risk of hyperemic injury caused by excessive cerebral vasodilation in a patient with impaired autoregulation has not been assessed.

## NEUROLOGIC AND NEURODEVELOPMENTAL OUTCOMES AFTER CARDIAC SURGERY WITH CPB

Neurologic injuries sustained during surgery with CPB can have long-term consequences. McQuillen *et al.* prospectively compared preoperative and postoperative MRIs in new-borns undergoing surgery for CHD. Of 53 newborns, 36% had new brain damage on MRI that was acquired either intraoperatively or shortly after surgery. Newborns with abnormal MRIs before surgery were not more likely to show brain damage after surgery. Postoperative cerebral injury was significantly more common in children who received the Norwood procedure. Postoperative brain damage included focal strokes and other moderate-to-severe injuries in both superficial and deep white matter. Factors associated with postoperative brain injury included lower bypass flow rates, worse intraoperative metabolic acidosis, retrograde cerebral perfusion, and low MAP on the first postoperative day [10]. Sarajuuri *et al.* also conducted MRI studies of children with single-ventricle heart disease and reported that watershed areas were particularly vulnerable to ischemia in the perioperative period [27].

Schmitt *et al.* reported alarmingly high rates of postoperative acute and permanent neurologic complications in children with CHD, including tetralogy of Fallot, ventricular septal defect, tricuspid atresia, anomalous pulmonary veins, and atrial septal defect [28]. None of these children had any known neurologic abnormalities preoperatively. Children with surgically corrected CHD are more likely to perform poorly on neurodevelopmental tests and require remedial educational services compared to healthy children, particularly if longer periods of bypass and circulatory arrest (CA) were required during surgery [3,4,6,8,27,29-31]. Children who underwent TGA repairs had neurodevelopmental delays regardless of whether the surgery was performed with DHCA or low-flow CPB [3,6]. Patients with single-ventricle physiology are at particularly high risk of impaired neurodevelopment, as the cumulative long-term effects of CPB and CA duration over staged surgical repairs are unclear [4,27,29].

## NEUROLOGIC MONITORING

Most neurocognitive evaluations for children who undergo surgery for CHD occur years after the surgery, when learning disabilities and behavior problems manifest at school or home. Due to the complexity and plasticity of the neonatal brain, signs of neurologic injury may not become evident until years after the perioperative insult [23]. Because neurologic events become irreversible in a time course that is measured in minutes, neurologic monitoring must be continuous and have a time resolution measured in seconds to effectively guide management. Such neurologic monitoring can assist clinicians in immediately identifying potentially catastrophic events during surgery. Emergencies such as kinked bypass hoses, malpositioned or slipped cannulae, and embolic events can be detected by sudden changes in neuromonitoring.

Currently, cardiopulmonary parameters are routinely monitored throughout cardiac surgery. However, standards for brain monitoring have yet to enter the cardiac operating room. Maintaining cerebral oxygenation and supporting cerebral autoregulation during the entire course of surgery and postoperatively are the goals of care for these children. Effective monitoring would improve our ability to target homeostatic regulation in the brain, decrease the neurologic injury inherent in CPB and DHCA, and result in improved functional outcomes [23]. Table **2** provides a summary of the advantages and limitations of the most commonly used, noninvasive neuromonitoring techniques during CPB.

### Near-Infrared Spectroscopy

Near-infrared spectroscopy (NIRS) is a commercially available, relatively inexpensive, and noninvasive technique that measures cerebral oxygen saturation. Oxygen consumption is considered one of the most reliable measures of brain metabolic activity [32]. NIRS measurements reflect the ratio of oxygen supply to extraction and correlate with tissue PO_2_ [33]. In piglets and rabbits, cerebral oximetry correlates with cellular indicators of cerebral metabolic activity [21,34].

The cerebral oximeter probes are placed on either side of the forehead with adhesive. The device uses differential near-infrared light absorption (700–900 nm) by oxygenated and deoxygenated hemoglobin. Measured absorption at the different wavelengths is converted to calculate oxyhemoglobin and deoxyhemoglobin concentrations with a modified Beer-Lambert equation. These devices calculate a regional cerebral oxygen saturation that reflects a combination of roughly 70% venous, 5% capillary, and 25% arterial cerebral blood volume. Thus, cerebral oximetry represents a mixed vascular saturation with bias toward the venous saturation. Cerebral oximetry is the ratio of oxyhemoglobin to total hemoglobin in the NIRS light path and is reported on a scale from 15% to 95%. NIRS can also be used to extrapolate cerebral blood volume based on hemoglobin concentration.

Commercial NIRS monitors measure cerebral oximetry regionally in the frontal cerebral cortex, a known watershed area. Injury to deep areas of the brain that are also vulnerable to ischemia during DHCA, such as the basal ganglia [35], may not be detected by NIRS. Measured cerebral oximetry values in the same patient can differ if the probe is placed at different locations on the forehead. The probe’s position influences factors that can affect the NIRS photon pathlength and light scattering. Other factors that affect the accuracy of NIRS monitoring include the shape of the forehead, structures external to the brain, depth of the brain surface, and extracranial blood flow [36].

Two commercially available cerebral oximetry monitors are approved by the US Food and Drug Administration (FDA) for use in the United States. In the case of the INVOS monitor (Somanetics), NIRS is used to trend cerebral oximetry. Baseline parameters are set from the initial readings taken, and future measurements are compared against the baseline. The baseline saturation should therefore be determined early in the operative course and during a period of hemodynamic stability. The newly FDA-approved ForeSight monitor (Casmed) has not yet been marketed to the extent of the INVOS monitor. As the Fore-Sight has demonstrated a fixed relationship to cerebral venous oxygen levels, it has a specific FDA approval for monitoring absolute cerebral oximetry as opposed to trend monitoring [37]. The impact of this difference has not yet been declared and will require a more extensive deployment of the Fore-Sight monitor for determination.

Cerebral oxygen saturation is determined by numerous clinical factors. Improved oxygen delivery, increased cardiac output, cooling, and selective cerebral perfusion (SCP) during DHCA increase cerebral oximetry readings. Hypoxia, low arterial blood pressure (ABP), low hematocrit, and DHCA decrease cerebral oximetry readings [21,36,38,39].

In general, in acyanotic patients without intracardiac shunting who are breathing room air, baseline cerebral oxygen saturation is approximately 70%; in cyanotic patients, it is 40%–60% [38]. Compared to patients with normal cardiac physiology, cerebral oximetry is significantly lower in patients with left-to-right shunting both without cyanosis (i.e., atrial septal defect, ventricular septal defect without right ventricular outflow tract obstruction) and with cyanosis (i.e., ductus arteriosus–dependent systemic or pulmonary circulation, single-ventricle physiology with systemic-to-pulmonary artery shunts, and tetralogy of Fallot variants) [40]. Baseline cerebral oxygen saturations may be <50% in neonates with TGA [24].

Lower cerebral oximetry measurements correlate with poorer neurologic outcomes [38], and perioperative mortality increases with lower preoperative cerebral oximetry [40]. In infants with TGA, lower preoperative cerebral oximetry is significantly associated with lower post-bypass cerebral oximetry [24]. In animal models, low cerebral oximetry measurements are associated with neuronal cell dysfunction and death [38].

It is controversial whether NIRS can substitute for jugular venous bulb oxygen saturation (SjvO_2_). Cerebral oximetry with NIRS incorporates arterial oxygen saturation, whereas SjvO_2_ measures only venous blood. NIRS only measures regional surface cerebral oxygenation, whereas SjvO_2_ measures global venous blood that includes deep brain structures. However, a good correlation between cerebral oximetry and SjvO_2_ has been reported in children with CHD and in animal studies [21,41], including children who weigh <10 kg. Additional studies are needed to further define the reliability of NIRS compared to SjvO_2_.

Mixed venous blood saturations (SvO_2_) are often used to assess cardiac output. Post-bypass trends in cerebral oximetry and SvO_2_ correlate in children with acyanotic and cyanotic heart diseases and in animal studies [42,43], although this relationship is inconsistent. Trends in cerebral oximetry and venous blood saturation from right atrial catheters also correlate [44], but Li *et al.* showed that following the Norwood procedure, the cerebral oxygen saturation only loosely correlated with direct measures of cardiac output and oxygen supply [43].

### NIRS During Bypass

NIRS trends are similar in neonates, infants, and children during bypass [45]. In the absence of cardiac mixing or shunting lesions, cerebral oximetry shows characteristic patterns during CPB and DHCA. During cooling, cerebral oximetry increases [21,22], presumably due to slowing cerebral metabolism with lower oxygen consumption. When bypass is initiated, cerebral oximetry decreases as blood pressure and hematocrit fall [24]. Changes in blood gas and surgical manipulation of the heart and great vessels are also likely important determinants of cerebral oxygenation at the start of CPB [34].

When DHCA is initiated, cerebral oxygenation continues to decline until it reaches a nadir. The slow decline in cerebral oxygenation during this time suggests that cerebral tissues continue to extract oxygen from the blood despite deep hypothermia after CA has already started [21,34]. The nadir in cerebral oxygenation that is eventually reached likely reflects the stop in cerebral perfusion and cessation of cerebral oxygen consumption. During retrograde low-flow perfusion (RLFP), however, cerebral oxygenation may increase to greater than the baseline level obtained before surgical incision [38].

With reperfusion and warming, cerebral oxygenation initially rises and then falls [21,22]. The initial rise in cerebral oxygenation reflects renewed oxygen supply to cerebral tissues. Cerebral oxygenation subsequently decreases as cerebral metabolic activity resumes with increasing oxygen consumption.

Toet *et al.* studied neonates with TGA who underwent arterial switch repair. The average preoperative cerebral oxygenation was <45%. Cerebral oxygenation increased to >55% during CPB, then declined to <50% post-bypass. Eventually, postoperative cerebral oxygenation reached the normal values that would be expected in patients without mixing cardiac lesions, but it took a surprisingly long time for cerebral oxygenation to achieve normal levels [24]. This pattern with a low post-bypass cerebral oxygenation has also been described after stage 1 palliation for HLHS [20].

Despite normal arterial oxygenation and normal blood pressure, some patients with repaired TGA did not reach normal cerebral oxygenation for more than 24 hr after surgery. Neonates who had lower preoperative cerebral oxygenation and longer periods of CPB and DHCA took longer to achieve normal cerebral oxygenation. Because hematocrit and cerebral blood volume remained unchanged, the authors attributed this extended period of low cerebral oxygenation to increased cerebral oxygen consumption following bypass. The authors theorized that this may have represented a metabolic response to hypothermia involving “restoration from oxygen debt,” or a response to brain rewarming. A chronic oxygen deficit before surgery could have caused higher oxygen utilization postoperatively [24]. Increased cerebral vasoconstriction has also been speculated to cause the observed low cerebral oxygenation after bypass during stage I palliation for HLHS [20].

The possible combination of postoperative vasoconstriction and increased CMRO_2_ raises concern that children with cyanotic heart disease are at higher risk of post-bypass cerebral ischemia than are children with normal cardiac physiology. Cerebral oximetry promises to enable physicians to better balance oxygen delivery with increased oxygen demand in real time.

### Using NIRS to Guide Therapeutic Interventions

McQuillen *et al.* studied 16 newborns with TGA, HLHS, or aortic coarctation. They performed NIRS monitoring and compared preoperative and postoperative MRIs. After surgery, six patients (two with TGA, four with HLHS) had MRI evidence of new brain injuries that were acquired intraoperatively or shortly after surgery. Compared to uninjured patients, the injured newborns had significantly lower mean cerebral oximetry levels during aortic cross-clamping and lower NIRS oxygenation trends after clamp removal and following CPB. Low bypass flow rates were significantly associated with postoperative brain injury, and four of these six patients received retrograde cerebral perfusion. This study is one of the first and largest to demonstrate an association between low NIRS saturation and radiographic evidence of brain damage following CPB [10]. These results also suggest that NIRS monitoring may be useful during retrograde cerebral perfusion to titrate flow rates.

Andropoulos *et al.* proposed a treatment algorithm for changes in cerebral oximetry [38]. We have provided a table based on similar principles (Table **3**). During the pre- or post-bypass periods, if cerebral oximetry decreases by ≥20% from a stable baseline, the anesthesiologist should consider maneuvers to increase the cardiac output, raise the hematocrit, raise inspired oxygen concentration, or increase PaCO_2_ to decrease cerebral vasoconstriction. If the absolute cerebral oximetry is <30%, immediately initiating or returning to bypass with aggressive efforts to improve oxygenation is indicated.

During bypass, if cerebral oximetry decreases by ≥20% from baseline, the anesthesiologist and perfusionist should consider adjusting the bypass flow rate, PaCO_2_, blood pressure, temperature, or hematocrit. The surgeons should check that the cannulae are positioned properly for optimal CBF. Migration or occlusion of the cannulae may be detected by a sudden decline in the cerebral oxygen saturation [46-48] while arterial pressure remains unchanged [49]. If the absolute cerebral oximetry is ≤30% or is at its nadir for more than 30 min, measures to improve cerebral oxygen delivery by adjusting bypass flow rates, PaCO_2_, blood pressure, temperature, hematocrit, or cannulae position are indicated. If absolute cerebral oximetry measures ≥95%, CBF velocity and bypass flow should be examined to avoid hyperperfusion injury. Hypercapnia may also cause cerebral vasodilation with abnormally high cerebral oximetry. During DHCA, if cerebral oximetry decreases to 30%, reperfusion is recommended.

**Fig. (1):**
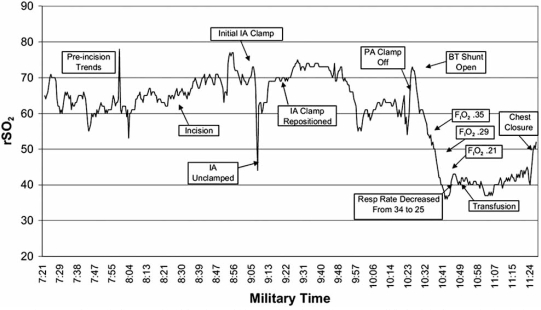
NIRS tracing over time of clamp malposition in an infant undergoing off-pump modified Blalock-Taussig (BT) shunt placement. Note the acute decrease in cerebral oximetry values (rSO_2_) with placement of the clamp on the innominate artery (IA) and prompt recovery with unclamping [51].

Bilateral cerebral NIRS monitoring can detect differential perfusion pressures or oxygenation status between hemispheres [47,50], which would be particularly important in a patient without an intact Circle of Willis during SCP techniques. Five percent of newborns do not have an intact circle of Willis [50]. In addition, multiple case reports demonstrate the utility of NIRS to detect cannula and clamp malposition during congenital heart surgery (Fig. **1**) [51].

Andropoulos *et al.* studied nineteen neonates undergoing aortic arch repair with antegrade RLFP and bilateral NIRS monitoring. Pre-bypass saturations were similar between cerebral hemispheres, but during the initiation of CPB, during RLFP, following RLFP, and after bypass, left-sided cerebral saturations were significantly lower than in the right hemisphere. In almost half of patients, the discrepancy between cerebral hemispheres was >10%. Interestingly, the CBF velocities measured by ultrasound remained similar between hemispheres, suggesting that the patients had complete circles of Willis. The unchanged CBF velocities also indicated that flow rates remained adequate, although it is difficult to draw conclusions because changes in cerebral vascular resistance were not measured. Decreased cerebral drainage with accumulation of desaturated venous blood in the left hemisphere may have been responsible for the lower left-sided cerebral oximetry [52].

NIRS technology can also measure splanchnic oxygenation during cardiac surgery and in the intensive care unit. Both cerebral and splanchnic oxygen saturations can be used to assess end-organ perfusion. Splanchnic oxygen saturation roughly correlates with general tissue oxygen delivery, although the correlation does not appear as strong as that seen with cerebral oxygen saturation [43]. The kidneys normally have a low oxygen extraction ratio, and perfusion is regulated primarily by sympathetic tone. In contrast, cerebral perfusion is closely coupled to metabolism [20].

When they are applied simultaneously, the renal saturation can serve as a control for the cerebral saturation by differentiating changes in total-body perfusion from changes in only cerebral perfusion and cerebral metabolic activity. Simultaneous decreases in renal and cerebral saturations indicate decreased total-body perfusion with impaired cerebral autoregulation. During periods of stress, the splanchnic saturation may be lower than cerebral saturation, as increased sympathetic tone to the kidneys re-distributes perfusion preferentially to the brain. A decrease in cerebral saturation without a change in renal saturation indicates that only cerebral perfusion and/or oxygen extraction have changed, which may occur secondary to cerebral vasoconstriction. For example, following stage 1 palliation for HLHS, post-bypass cerebral oxygenation decreases more than splanchnic saturation, suggesting that cerebral vasoconstriction increases after CPB in these patients, thereby reducing cerebral oxygen delivery and increasing the risk of brain ischemia [20].

### Limitations of NIRS

Critics of cerebral oximetry cite wide inter-patient variability, different readings from different areas on the same patient’s forehead, the inability to measure global cerebral saturation, and the absence of a universal “normal” value as limitations. The oximetry readings depend upon CBF, hematocrit, and oxyhemoglobin. If CBF and hematocrit remain constant, oximetry values over time can be compared with respect to venous and arterial oxygen content. However, if CBF swings passively with changes in CPP and MAP, such as when cerebral pressure autoregulation is lost during hypothermic CPB or when MAP crosses the autoregulatory thresholds, changes in NIRS will reflect more than just the cerebral oxygenation status.

NIRS cerebral oximetry is calculated based on an algorithm that assumes that 75% of the cerebral blood volume is venous and the other 25% is arterial. This assumption is based on adult data and has not been verified in pediatrics. Some patients have high cerebral oximetry readings of almost 95% during hypothermic bypass. As the maximal cerebral oximetry value is 95%, cerebral hyperperfusion may be poorly detected by NIRS alone. Hyperemia can result in intracranial hemorrhage and cerebral edema [53]. NIRS monitoring can be used to titrate flow rates during SCP. However, NIRS monitors only regional, frontal cerebral cortex. It does not measure oxygenation of deep brain structures. Raising flow rates too high in attempts to increase the NIRS value may cause hyperperfusion injury in deeper brain structures.

## ELECTROENCEPHALOGRAM

Electroencephalogram (EEG) monitoring has been used in the cardiac surgical setting to titrate cooling strategies, detect intraoperative events, and monitor for seizures postoperatively. The EEG is affected by anesthetic agents, temperature, and CPB. In most patients, EEG is isoelectric during CPB [24]. With DHCA, waiting to achieve an isoelectric EEG during cooling before initiating CA is potentially neuroprotective [54]. The degree of hypothermia required to induce electrocerebral silence varies greatly among patients. Temperatures at which electrocerebral silence on EEG occurs are lower than when evoked potentials disappear, making EEG a more reliable method of neuromonitoring during cooling [55].

EEG activity resumes upon cerebral reperfusion and rewarming. The temperature at which continuous EEG activity returns is predictive of postoperative neurologic dysfunction as this appears to be related to intraoperative brain injury. In adult studies, the risk of postoperative confusion or stroke significantly increases with every degree higher at which continuous EEG activity resumes [56].

Postoperative EEG monitoring detects seizures that clinically would be missed due to pharmacologic neuromuscular blockade and sedation, including subclinical seizure activity [28]. Seizures may occur secondary to cerebral injury and worsen pre-existing or ongoing neuronal damage. Gaynor *et al.* performed EEG monitoring on 183 infants after cardiac surgery. Eleven percent of the patients had EEG-detected seizure activity postoperatively; none of these children had clinically apparent seizures. Children with HLHS were significantly more likely to have postoperative seizures than children with TGA, tetralogy of Fallot, ventricular septal defect, coarctation, other two-ventricle defects, and single-ventricle defects other than HLHS. Seizures were also significantly more likely with longer periods of DHCA. The increased risk of seizures following surgery for HLHS was likely related to prolonged DHCA. However, some infants who received bypass without DHCA or who had short periods of DHCA still had postoperative seizures [57].

Postoperative diffuse EEG slowing has been reported in children during the first 48 hr following cardiac surgery. However, EEG slowing was not associated with neurologic deficits. EEG slowing was actually more common in children who had shorter periods of CPB and lower levels of markers for inflammation and oxidative stress [28].

### Bispectral Index Monitoring

Conventional EEG can be technically difficult to use and interpret in the operating room. However, limited-channel, EEG-based technologies with automated interpretation correlate electrical brainwave activity with depth of anesthesia and awareness. Commercially available devices are the Bispectral Index (BIS) monitor (ASPECT Technologies, USA) and, most recently, the SED line monitor (Hospira, USA). These monitors use algorithm-based analysis of multiple EEG characteristics and integrate them into a single, dimensionless number. Unlike routine EEG, these monitors utilize placement of a single sensor that contains multiple electrodes, which are positioned easily on the forehead and temple in a specific pattern. The BIS utilizes a unilateral sensor array, whereas the SED line utilizes a bilateral sensor. At this time, only the BIS has an approved pediatric probe. Though these monitors have directed FDA approval to assess anesthetic depth [58], there have been multiple reports of additional applications of the BIS monitor (i.e., burst suppression, sedation monitoring) throughout the perioperative period. The BIS index ranges from 0 (isoelectric EEG or electrocerebral silence) to 100 (awake). A BIS reading of ≥80 prompts the anesthesiologist to examine the anesthetic depth and risk of awareness under anesthesia. BIS decreases with deeper hypothermia during bypass [59]. The BIS monitor’s utility has not been well tested in children younger than 2 years of age.

During cardiac surgery, BIS monitoring has been reported to detect brain ischemia in real time. A sudden decrease in the BIS reading has been suggested to indicate cerebral ischemia, especially when correlated with specific events in the surgery, such as the initiation of CPB when acute hemodilution and decreasing MAP and CPP reduce oxygen supply to the brain [19,58]. However, in a study of neonates undergoing TGA repair, EEG activity did not differ with hypothermia or duration of CPB or DHCA, despite persistently low cerebral oximetry [24]. It should be noted that the interpretative algorithms of the BIS are designed for the detection of awareness—not ischemia.

Modeling routine EEG applications, the BIS monitor has been used to identify burst suppression or electrical silence to help determine when the brain is adequately cooled and cerebral metabolism is sufficiently slowed before initiating DHCA. Variability among patients in the degree of hypothermia that induces electrocerebral silence [55] makes neuromonitoring important. BIS decreases to zero with deep hypothermia, although the rate of decrease varies among patients [60]. A relatively high BIS reading suggests that further cooling is necessary before starting CA. Inadequate or non-homogenous brain cooling before CA increases the risk of neurologic injury [61].

The BIS monitor can also measure depth of anesthesia throughout surgery and during rewarming [62,63]. For instance, a BIS reading of <30 suggests that too much volatile agent is being delivered through the bypass pump. However, BIS readings are not changed by benzodiazepines and opioids. Therefore, a BIS reading of >80 during rewarming does not necessarily imply awareness under anesthesia if benzodiazepines are administered. After DHCA, the rate of BIS increase during rewarming varies among patients and is related to the duration of DHCA [60]. Delayed return of BIS activity during rewarming may be due to derangements in cerebral metabolism [64].

### Transcranial Doppler Ultrasonography

Pulsed-wave transcranial-Doppler (TCD) ultrasonography is a real-time method of measuring cerebral arterial blood velocity, which can be used as a surrogate for CBF. Like NIRS and BIS, TCDs are noninvasive and carry minimal risks to the patient. They can be used to assess whether cerebral autoregulation remains intact across changes in blood pressure and temperature. Age-specific normals values in TCD measurements are listed in Table **4** [65-67].

TCD does not assess absolute CBF, and calibrating velocity to flow is not possible due to the dynamic nature of the cerebral vasculature. However, during hypothermic CPB changes in CBF velocity are thought to correlate well to changes in CBF [50,53]. As flow velocity in the middle cerebral artery has been used with great success as a surrogate of CBF in quantifying cerebrovascular autoregulatory responses [68,69], the assumption is generally accepted.

Typically, the patient serves as the control, and measurements are weighed against the patient’s baseline obtained prior to surgical incision. Most commonly, the middle or anterior cerebral arteries are monitored. In infants, the TCD probe can be placed over the anterior fontanel to assess the internal carotid artery. The threshold flow rates for minimal cerebral perfusion during low-flow CPB or low-flow SCP can be identified. However, accurately measuring TCDs of a specific cerebral artery during bypass without pulsatile perfusion is technically challenging. Like NIRS monitoring, TCDs assess only one region of the brain.

Hemodynamic instability, general anesthesia, and low temperature decrease Doppler velocities. Reduced velocities during cooling may be attributed to lower cerebral metabolic demand with concomitant decreased blood flow. Lesions that cause a large diastolic run-off, such as a large patent ductus arteriosus, also decrease cerebral diastolic blood flow.

The role of TCD in a neuromonitoring algorithm is included in Table **3**. Pre- and post-bypass, deviations in the mean CBF velocity by ≥25% from baseline indicate potentially clinically significant changes in cerebral perfusion. Hypo- or hypercapnia cause cerebral vasoconstriction or vasodilation, respectively. Too low or too high systemic blood pressure may cause brain hypo- or hyperperfusion if cerebral autoregulation is impaired. During bypass, a change of ≥25% from baseline should prompt the anesthesiologist to check the bypass flow rates, PaCO_2_, blood pressure, anesthetic depth, and cannulae positions. TCD ultrasonography can also detect cerebral emboli as high-intensity transient signals with characteristic visual and audio signals.

### Multimodal Neurologic Monitoring

Each neurologic monitoring modality has its strengths and limitations (Table **2**). For instance, although NIRS is technically easy to use, it only measures oxygen levels in regional, superficial, cerebral structures. Simultaneous multimodal neurologic monitoring during cardiac surgery may provide more sensitive neurophysiologic assessments. In theory, an expansion of monitoring modalities decreases the time to detection and correction of deleterious changes in cerebral perfusion and oxygenation.

Concurrent TCDs and cerebral oximetry are being used in pediatric cardiac operating rooms. Combining TCD ultrasonography of the middle or anterior cerebral arteries and frontal lobe, NIRS allows continuous, real-time monitoring of up to 70% of hemispheric blood flow. During hypothermia, cerebral hyperperfusion may be detected by simultaneous increases in CBF velocity and cerebral oximetry.

In a study by Andropoulos *et al.*, neonates undergoing the Norwood procedure for HLHS or aortic arch reconstruction for interrupted or hypoplastic aortic arch received RLFP. Patients were monitored with both NIRS and middle cerebral artery TCDs. Baseline TCDs were measured during full-flow bypass. The flow rate during RLFP was then titrated to keep the TCDs within 10% of this baseline value. Several patients had very high cerebral oximetry measurements of 95% during RLFP. Because the maximal cerebral oximetry reading is already 95%, NIRS alone could not have detected cerebral hyperperfusion. However, measuring CBF velocity with TCDs enabled detection of hyperperfusion, theoretically reducing the risk of cerebral edema and intracranial hemorrhage [53].

When NIRS readings are not high during RLFP, some practitioners will increase perfusion flow rates in attempts to raise the cerebral oximetry reading, but correlation is poor between perfusion flow rate, cerebral oximetry, and CBF velocity during RLFP. Raising the perfusion flow rate can cause cerebral hyperperfusion that may only be detected by TCD ultrasonography [53].

Simultaneous BIS and NIRS monitoring has also been employed to identify ongoing brain ischemia. A decline in BIS reading could indicate either deeper anesthesia or cerebral ischemia, and the lower limit for cerebral oximetry when ischemia occurs is unknown. However, concordant decreases in both cerebral oximetry and BIS may be more suggestive of cerebral ischemia [19,58]. Combining somatosensory-evoked potentials and cerebral oximetry has also been explored in adults [70].

Multimodal neuromonitoring may be expensive and somewhat technically challenging. It would be difficult to fit a cerebral oximetry probe, a TCD probe, and EEG leads or BIS electrodes onto a small infant’s head. Adding three or more additional monitoring parameters to the cardiac operating room and quickly interpreting the data in a meaningful way could prove challenging for even the most vigilant anesthesiologist.

## DECREASING NEUROLOGIC MORBIDITY DURING CPB

A variety of techniques is employed intraoperatively to protect infants and children who undergo CHD repair against neurologic injury. Neuromonitoring, in conjunction with controlled hypothermia, anti-inflammatory strategies, and improved cerebral perfusion strategies, has become routine in the care of these children. The most prevalent neuroprotective care practices used during CPB for children with CHD will be discussed.

### Hypothermia

Hypothermia during CPB provides neuroprotection by decreasing cerebral metabolism. However, evidence suggests that cerebral oxygen consumption continues for a time period despite even deep hypothermia [21,22]. The oxygen-carrying capacity of hemoglobin declines during hypothermia [71], and this may be worsened by hemodilution from CPB [72,73]. Cerebral autoregulation is impaired at profound hypothermia [50] and during CPB [18]. Brain ischemia may still occur despite hypothermia when low MAP causes cerebral hypoperfusion while cerebral oxygen demands persist.

The intense catecholamine release from surgical stress may also counteract the protective effects of hypothermia on cerebral metabolism. When the blood-brain barrier is intact, catecholamines do not affect cerebral metabolism. However, hypothermia alters blood-brain barrier permeability and may permit catecholamines to increase cerebral metabolism [32]. Younger children may be more susceptible to this effect, as infants and young children with CHD have demonstrated higher levels of circulating catecholamines both before and during bypass, when compared to older children with cardiac disease [74].

### Deep Hypothermic Circulatory Arrest

CA places the brain at significant risk of injury from hypoperfusion and global ischemia. DHCA employs deep hypothermia to slow cerebral metabolism, decrease oxygen demand, and provide neuroprotection. Deeper hypothermia results in greater suppression of cerebral metabolism [75].

Although DHCA decreases the likelihood of brain injury from CA without deep hypothermia, DHCA itself still carries significant neurologic risks. Piglets suffer diffuse brain injury from DHCA [76]. The cessation of cerebral perfusion and the systemic inflammatory response induced by DHCA play key roles. Longer periods of DHCA are significantly associated with postoperative neurologic dysfunction [55] and negatively affect neurocognitive outcomes in children [3,77,78]. Generally, 30–40 min of DHCA at 18°C is considered a “safe” period for pediatric heart surgery [35]. For infants with TGA, IQ scores significantly decline once CA exceeds 42 min during the arterial switch procedure [3,78].

It is crucial that cerebral metabolism is fully halted before CA is initiated. Inadequate cooling or non-homogeneous cerebral cooling before initiating CA place the child at risk of neurologic injury [8]. There is also concerning evidence that cerebral metabolism with continuing oxygen requirements persists despite deep hypothermia during the beginning phase of DHCA [21,22], thus placing the brain at extremely high risk of ischemia, as the brain still requires oxygen but perfusion has stopped.

The brain is also subject to ischemia-reperfusion injury after DHCA. Even when perfusion is restored, cerebral metabolism, oxygen extraction, and pressure autoregulation remain impaired [61,75,79]. Disrupted cerebral autoregulation and metabolism can persist for 24 hr after DHCA [80]. Piglet studies show that cerebrovascular and oxygenation responses to hypoxemia and hypercapnia are impaired following DHCA. Altered CBF after DHCA may be mediated in part by inflammation [39,79,81].

### Low-Flow CPB

Low-flow CPB may decrease neurologic complications compared to DHCA, including decreased risk of seizures, by providing a continuous, low supply of oxygen to the brain [12]. However, cerebral hypoperfusion and ischemic injury can still occur. In piglets, low-flow CPB causes selective neuronal necrosis in the neocortex, hippocampus, basal ganglia, thalamus, white matter, and cerebellum [82].

The Boston Circulatory Arrest Trial was a single-centered trial involving infants with TGA. The infants underwent arterial switch correction by 3 months of age after randomization to DHCA or low-flow CPB at 0.7 L/min/ m^2^ (50 mL/kg/min). Infants in both groups had some period of CA during atrial and ventricular septal defect repairs. Neurodevelopmental outcomes were tested at 8 years of age in 155 patients. Compared to children who had low-flow CPB, the children who received total DHCA scored lower on tests for motor function, verbal skills, visual-motor tracking, and phonologic awareness (a skill important for reading). However, longer bypass duration with low-flow CPB was associated with future impulsive behavior and school problems [3,30]. Hovels-Gurich *et al.* showed significant correlation between the duration of low-flow bypass at 0.7 L/min/ m^2^ and neurologic and speech dysfunction in children 8–14 years old who underwent TGA repair as neonates [6]. Thus, while low-flow CPB can potentially improve neurologic outcomes by decreasing cerebral ischemia from CA, it may also contribute to neurocognitive dysfunction by prolonging the bypass duration.

Combining DHCA and low-flow bypass techniques may better support cerebral metabolism. Intermittent low-flow CPB during DHCA improves brain oxygenation in piglets [35]. Deep hypothermic continuous low-flow (DHCLF) bypass may also be useful. As previously reviewed, cerebral oxygen requirements may persist despite deep hypothermia. To manage this need, DHCLF provides some oxygen supply to reduce the risk of cerebral ischemia. NIRS monitoring during DHCA, low-flow CPB, or a combination of these techniques is critical to continuously assess cerebral oxygen supply and demand in real time.

### Selective Cerebral Perfusion

SCP or RLFP mitigates cerebral hypoperfusion injury associated with DHCA during pediatric surgery [50]. By providing higher and continuous CPP, some advocates believe that SCP offers better cerebral protection than does hypothermic CPB or DHCA alone. In pigs, SCP appears to better support cerebral metabolism than does CPB, but CBF gradually declines with prolonged SCP [32]. Schears *et al.* demonstrated improved cortical oxygenation in pigs with SCP as compared to DHCA alone [13].

If cerebral autoregulation is impaired, the pressure-passive nature of CBF places the brain at risk of hyperperfusion injury, cerebral edema, and intracranial hemorrhage from high SCP flow rates [13,20,50]. Cerebral hyperperfusion may be detected through NIRS and TCD monitoring. Multimodal neuromonitoring can assist in titrating flow rates to provide optimal CBF.

**Fig. (2):**
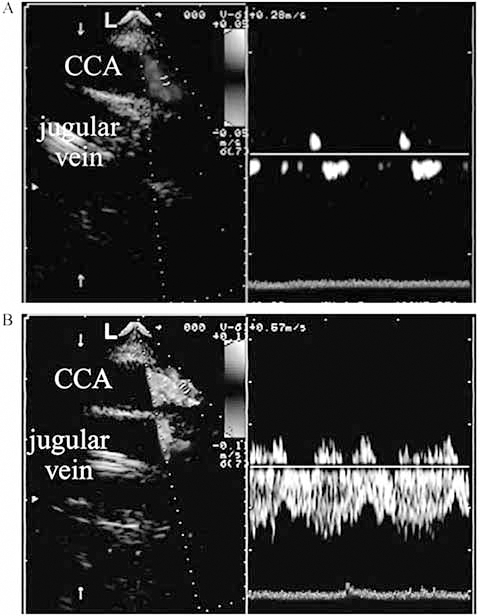
CBF abnormalities detected by multimodal neuromonitoring. Transesophageal echocardiogram showing catheter malposition (**A**) and correlation with measured flow velocity in the right common carotid artery (CCA). Note the removal of obstruction and return of flow after catheter repositioning (**B**) [48].

By using a retrograde approach to perfuse the brain, it is difficult to measure the amount of cerebral perfusion that is actually achieved. Competent valves in the internal jugular venous system can impede retrograde flow, although blood flow may still reach the brain through collateral veins [83]. McQuillen *et al.* performed MRI studies on newborns with CHD. Newborns who received retrograde cerebral perfusion were at higher risk of acquiring brain damage intraoperatively or shortly after surgery. Interestingly, focal strokes occurred in both hemispheres [10]. Antegrade regional low flow cerebral perfusion [48,84] may improve CBF by avoiding the valvular venous system. Piglets that receive antegrade RLFP have improved postoperative neurobehavior scores and less hippocampal injury compared to piglets that receive DHCA alone. NIRS, TCD, and BIS may be helpful in this setting to detect potentially catastrophic cannula malpositioning. The power of combining these observations demonstrates how CBF should be continuously assessed with neuromonitoring to ensure that the cannula has not slipped into a suboptimal position [48,84] (Fig. **2**).

### Pulsatile Flow with CPB

Nonpulsatile flow during CPB provides some tissue perfusion through nonphysiologic conditions. Pulsatile flow may provide a hemodynamic state that is closer to physiologic and may potentially improve oxygenation and better support metabolism. In piglets, pulsatile flow during CPB decreases cerebral vascular resistance, increases global CBF, and improves regional CBF to the cerebral hemispheres, cerebellum, thalamus, basal ganglia, and brainstem compared to piglets that receive nonpulsatile CPB at similar MAPs [85].

### Modulating Inflammation

CPB induces a significant, systemic inflammatory response that plays a key role in cerebral injury. Methods to decrease the inflammatory response to bypass include corticosteroids, aprotinin, modified ultrafiltration (MUF), zero-balance ultrafiltration (ZBUF), and leukocyte depleting filters. Pharmacologic interventions to target specific components of the complement and cytokine cascades are also being explored [2,86,87].

Contact between blood and the bypass circuit activates inflammation. Priming the circuit with blood products to avoid hemodilution in neonatal surgery further increases circulating levels of proinflammatory mediators, including cerebral TNF-α mRNA [79]. Heparin-bonded circuits may decrease this inflammation [2]. In neonatal piglets, a miniaturized, bloodless, primed, CPB circuit reduced the inflammatory response to DHCA. Interestingly, this circuit was also associated with improved CBF following DHCA, a phenomenon that the authors postulated was due to decreased inflammation [79].

DHCLF bypass with a miniaturized circuit and a bloodless prime may prove particularly beneficial. Clinically significant hemodilution, even for neonatal surgery, could be avoided with the small circuit. Combined with deep hypothermia, the continuous, low state of perfusion may decrease the risk of cerebral ischemia.

### Steroids

Steroids have been used for neuroprotection and to decrease inflammation in response to CPB and DHCA. Both dexamethasone and methylprednisolone have been studied during CHD repair and their effectiveness remains controversial. Gessler *et al.* reported that adding prednisolone to the CPB priming solution did not suppress the systemic inflammatory response to bypass in infants [88]. In piglets that undergo DHCA, methylprednisolone decreases cerebral edema, decreases neuronal death, and improves global and regional CBF with improved oxygen delivery to the cerebral hemispheres, cerebellum, basal ganglia, and brainstem [72, 89]. However, Schubert *et al.* found that methylprednisolone did not significantly affect CBF or brain edema after DHCA in piglets. In fact, the piglets that received methylprednisolone suffered more hippocampal neuronal apoptosis than piglets that did not receive steroids [90]. By decreasing inflammation, methylprednisolone may also suppress the neuroprotective and regenerative effects of certain interleukins, such as IL-6 [91]. The effects of steroids on cerebral autoregulation during CPB are unknown.

### Ultrafiltration

MUF decreases levels of circulating inflammatory mediators, lowers total body water, and raises the hematocrit after CPB in infants and neonates. It may improve neurologic outcomes by mitigating inflammation, decreasing cerebral edema, and improving oxygen delivery after CPB and DHCA [92, 93]. In piglets, MUF improves post-bypass cerebral oxygen delivery and cerebral metabolic oxygen consumption independent of higher hematocrit [94]. However, Myung *et al.* reported that MUF did not prevent neurologic injury or change neurologic outcomes after DHCA in piglets [95]. In fact, the effects of hemoconcentration after MUF may actually raise absolute levels of inflammatory mediators [93].

In ZBUF, the volume removed during filtration is replaced with a balanced salt crystalloid solution. ZBUF removes proinflammatory mediators without altering the patient’s volume status, thus avoiding a hemoconcentration effect that would raise absolute levels of inflammatory mediators. ZBUF appeared more effective in reducing circulating proinflammatory cytokines, including TNF-α, IL-6, and IL-8, compared to adding methylprednisolone to the bypass circuit prime. However, total body water was not decreased which may raise the risk of post-bypass cerebral edema [86].

### Vasodilator Therapy

Numerous vasodilators have been utilized in attempts to improve CBF rates during CPB. The success of these vasodilators in preventing brain injury is debatable. In neonates undergoing TGA correction, sodium nitroprusside appears neuroprotective and may decrease reperfusion injury during the 48 hr following bypass [96]. In pediatric patients, bypass flow rates with similar MAPs are higher with phenoxybenzamine than with sodium nitroprusside, resulting in greater oxygen delivery to vital organs [74]. However, Gazzolo *et al.* reported that phentolamine given to infants during the cooling and rewarming phases of CPB resulted in significantly higher levels of S100B, a possible marker for brain injury [97].

When cerebral autoregulation is impaired during hypothermic CPB, vasodilators can theoretically worsen hyperemic brain injury when bypass flow rates are too high or during post-CBP hyperemic responses. Vasodilators could also disrupt cerebral autoregulatory mechanisms, even when MAP is maintained within the cerebral pressure autoregulatory range. Further research on the effects of vasodilator therapy on cerebral autoregulation during CPB is needed.

Post-bypass, the remnant hypotensive effects of vasodilators place the brain at risk of hypoperfusion during periods of low MAP. For instance, the half-life of intravenous phenoxybenzamine is approximately 24 hr. Neurologic monitoring to ensure that the brain is receiving adequate blood flow and oxygenation in the postoperative period is essential. Ideally, monitoring to ensure that MAP remains within the cerebral autoregulatory range would guide blood pressure management.

Nagdyman *et al.* examined the effects of sildenafil on postoperative CBF in children with CHD. Levels of both oxygenated hemoglobin and total cerebral hemoglobin increased immediately after administration of sildenafil [98], suggesting that sildenafil caused an increase in cerebral blood volume, changed neuronal metabolism, or both. If sildenafil indeed has clinically significant cerebral vasodilatory effects, it would be important to monitor for cerebral hyperperfusion and impaired cerebral autoregulation, particularly during periods of hemodynamic instability in the postoperative period when sildenafil is used.

## FUTURE DIRECTIONS

As previously discussed, cerebral pressure autoregulation maintains constant CBF across changes in CPP. Cerebral autoregulatory mechanisms function within a specific blood pressure range. The high and low limits for MAP for cerebral autoregulation in pediatric patients are unclear. The thresholds probably differ according to gestational and chronologic age and type of heart disease. 

Impaired autoregulation results in a narrowed range of tolerable CPPs at which the brain can appropriately match CBF to metabolic needs. Outside of this range, CBF becomes passive to systemic and cerebral perfusion pressures, rendering the brain vulnerable to ischemia or hyperemic injury. Hypothermic CPB impairs cerebral autoregulation [18-20,50,99].

Autoregulation monitoring is fundamentally different from other forms of neuromonitoring: instead of monitoring and calibrating a signal of flow or oxygenation, an autoregulation monitor scans for low-frequency waveforms in flow or oxygenation that are transmissions of similar frequency waveforms of ABP. The finding of such a transmission is reported as pressure passivity by the monitor. Such an approach is a focus shift from defining ischemic thresholds to finding a range of ABP that optimizes autoregulatory responses. Absolute values of flow and oxygenation are irrelevant to this analysis, but obviously flow and oxygenation will be affected by ABP that is outside of the autoregulatory range. Autoregulation assessment is becoming increasingly recognized as an important aspect of the care of patients with traumatic brain injury [100,101], and the physiologic principles driving that paradigm shift are applicable to neurologic injury sustained during correction of congenital heart lesions. Traumatic brain injury is the logical birthplace of this technology, as it is the clinical niche for invasive neuromonitors used in autoregulatory assessment. Until recently, the practical limitations for the transfer of this modality to the cardiac care arena have been the invasive nature of the monitors (intracranial pressure monitors [102], laser-Doppler probes [103], and parenchymal tissue oxygenation monitors [104] or, in the case of noninvasive TD monitoring [68]), the technique can be too operator dependent to deploy, and too difficult to maintain during CPB, when the pulsatile signal is gone.

A new technique of monitoring autoregulation continuously applies the same waveform-analysis construct used in invasive monitors of autoregulation to the noninvasive signal of near-infrared cerebral oximetry monitoring. The assumption with this technique is that the cerebral rate of oxygen consumption is relatively static over each analysis period and, therefore, that changes in cortical oxygenation at the frequency of analysis are due to changes in CBF. These assumptions proved valid in a piglet model in which hypotension was induced and the LLA was accurately detected by cross-correlation of low-frequency ABP and cerebral oximetry waveforms in 300-second periods [105]. This index is called the cerebral oximetry index (COx), a name that reflects the phylogenetic relationship of the index with its predecessors, the pressure-reactivity index (PRx) and the mean-velocity index (Mx).

The PRx and Mx were the first continuous indices of autoregulation that detect the passive transmission of *spontaneous* waves of CPP to CBF surrogates. The analysis is performed in the time domain by means of a moving, linear correlation coefficient but is frequency specific because pulse and respiratory harmonics are first removed with a low-pass filter [68,102]. This method, originating at Cambridge University, obviates the need to induce an ABP change (i.e., tilt-table or thigh-cuff maneuver) to assess autoregulation, thereby allowing for *continuous* monitoring. Because passive wave transmission is quantified with a linear (Pearson) coefficient, the resultant index is a number between –1 and 1, where 1 indicates absolute pressure passivity, 0 indicates no transmission, and –1 indicates absolute pressure reactivity. The normal range of the index depends on the parameter used in the correlation: The intracranial pressure should demonstrate pressure reactivity, and a normal PRx is negative, but surrogates of CBF should demonstrate no transmission from slow waves of ABP, and normal values of the COx and Mx are zero.

Regardless of which of these indices is used, increasingly positive values are increasingly pathologic, indicating pressure passivity. Loss of autoregulation detected by these indices is associated with poor outcome in patients with traumatic brain injury [106], and, in premature neonates, it is associated with intraventricular hemorrhage and periventricular leukomalacia [107-109]. Continuous monitoring of autoregulation can identify an optimal range of blood pressure that poses minimal challenge to the autoregulatory mechanism, and management outside this range has an association with poor outcome following traumatic brain injury [101].

**Fig. (3):**
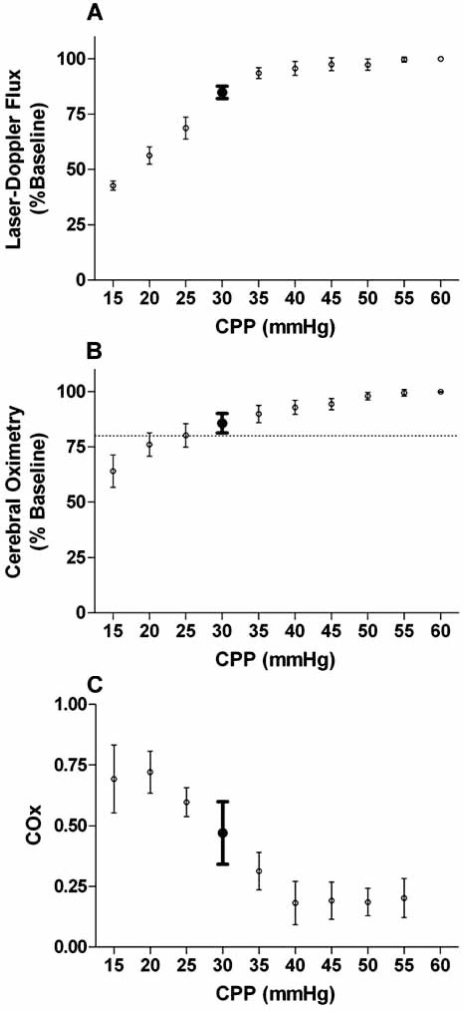
Comparison of (A) cerebral blood flow, (B) simple cerebral oximetry, and (C) the cerebral oximetry index (COx). The COx is a continuous monitor of autoregulation derived from cerebral oximetry. Data from 6 piglets with progressive hypotension are shown, with measurements of laser-Doppler flux, cerebral oximetry, and COx plotted as a function of cerebral perfusion pressure (CPP). A 20% reduction in cerebral oximetry is seen at a CPP of 25 mmHg [horizontal dashed line in (B)], well below the lower limit of autoregulation (LLA) at a CPP of 35 mmHg (shown by bold error bars in all three graphs). In contrast, the COx increase is easily detectable at the LLA.

Because the COx uses cerebral oximetry as a surrogate of CBF, it is not invasive or operator dependent. This modality is therefore practically suited to the congenital cardiac arena to define neuroprotective blood pressure ranges for individual patients, and studies are currently underway to determine the impact of this monitoring. We have found in our piglet studies that simple cerebral oximetry is not sensitive to loss of autoregulation. Piglets have values of cerebral oximetry that are still within 20% of baseline values when their blood pressure is well below the lower limit of pressure autoregulation [105]. Cerebral oximetry does detect critically low blood pressures when frank ischemia is imminent in these studies. We suggest that NIRS is an excellent indicator of critical neurologic events and that critically low values are specific indicators of abnormality. However, normal values are not necessarily reassuring of optimal care parameters. When cerebral oximetry was used to quantify pressure passivity with the COx, we found that the result was much more sensitive to loss of autoregulation, even before critically ischemic conditions were present (see Fig. **3**).

Patients with single-ventricle heart disease are at particularly high risk of neurologic injury from altered cerebral autoregulation. The bidirectional, cavopulmonary anastomosis relies on venous drainage to drive pulmonary blood flow. In neonates, the venous pressure that determines this upstream gradient largely comes from cerebral drainage [110]. However, high anastomosis pressures could impede cerebral venous drainage, thus altering CPP, which could theoretically shift the cerebral autoregulatory range. With the COx, these patients’ cerebral vasoreactivity could be continuously and easily monitored. Medical practitioners could then keep the patient’s MAP within the patient’s ideal autoregulatory range to support cerebral metabolic needs.

The COx is ideally suited for patients undergoing cardiac surgery. It is noninvasive, technically easy to use, has minimal-to-no risks, and holds promise to improve the care of pediatric patients in whom the cerebral autoregulatory range for blood pressure remains a mystery.

## CONCLUSION

Numerous preoperative, intraoperative, and postoperative factors place children at risk of neurologic injury while they undergo surgical repair of CHD. In particular, CPB adversely affects the brain through a variety of mechanisms. Injuries sustained during bypass or in the immediate postoperative period can have lifelong consequences. Some of these neurologic insults can be decreased through modulating inflammation, hypothermia, and regional perfusion techniques. However, despite these improvements in bypass protocols, CPB still carries significant risk of neurologic damage. Neuromonitoring to detect impending or ongoing cerebral ischemia can enable care providers to adjust clinical parameters to better support cerebral metabolism in real time. Vasoreactivity indices, particularly the COx, may prove useful in the future by supporting cerebral autoregulation in the perioperative period.

## Figures and Tables

**Table 1a:** Preoperative Risk Factors for Perioperative Cerebral Injuries and Poor Neurodevelopmental Outcomes in Children with CHD

Risk Factor	Examples
Genetic syndromes associated with abnormal neurodevelopment	Trisomy 21, velocardiofacial syndrome, DiGeorge, Turner,Williams, Alagille, CHARGE, and VACTERL [7]
Abnormal *in utero* neurodevelopment	Delayed myelination, immature sulcation, decreased parenchymal volume [10]
Congenital CNS abnormalities	Intracranial venous and cystic malformations [10]
Complicated pre-operative course	Prematurity, IUGR, sepsis, mechanical ventilation, hemodynamic instability, persistent hypoxia with arterial saturation less than 85% [6,9], persistent acidosis [6], IVH [6,7], poor glycemic control [1], hyperthermia [1], multiorgan dysfunction, and other comorbidities
Brain injuries acquired pre-operatively	Injuries involving both superficial and deep white matter, focal strokes [middle, posterior, and anterior cerebral artery distributions], hemorrhages [intraventricular, subependymal, subdural] [10]

CHARGE, coloboma, heart defect, atretic choanae, retardation of growth and development, genitourinary anomalies, ear abnormalities; VACTERL: vertebral anomalies, anal atresia, cardiac anomaly, tracheoesophageal fistula, renal and limb abnormalities; IUGR, intra-uterine growth retardation; IVH, intraventricular hemorrhage.

**Table 1b:** Risk Factors during CPB for Perioperative Cerebral Injuries and Poor Neurodevelopmental Outcomes

Risk Factor	Examples
Prolonged bypass time [9,5]	
Obstructed cerebral venous drainage decreases CPP and CBF	Improperly sized venous cannulae, malpositioned cannulae, insufficient drainage systems, small tubing, airlocks in the circuit [1]
Inflammation	Pediatric patients have a stronger inflammatory reaction to CPB than do adults due to a greater imbalance between proinflammatory and anti-inflammatory mediators [11], greater exposure of blood volume to the bypass circuit [86], and higher pump flow rates [2]. Activated neutrophils adhere to cerebral capillary beds, reduce CBF, disrupt the BBB, infiltrate into surrounding tissues, and cause cerebral edema with risks of elevated ICP. Leukocyte activation plays a pivotal role in ischemia/reperfusion injury during weaning from CPB and in the post-bypass period [11,87]. Significantly higher levels of intracerebral TNF-alpha mRNA have been reported following circuit priming with blood products [79]. Inflammation may also be partially responsible for impaired CBF after DHCA [79].
Embolic phenomena [1,11,12,111]	Thromboemboli [2,111], platelet-platelet aggregates [11], leukocyte-platelet conjugates [11], and microemboli from CPB circuit [including thrombus, air, lipid]. Thromboemboli may form despite heparinization with an ACT greater than 400 seconds [2,111]. Pediatric patients with CHD also have low baseline anti-thrombin III levels [111].
Hemodilution [14,71]	Anemia worsens cerebral oxygenation and increases the risk of ischemic injury [72,73].
Anticoagulation [40]	Intracranial bleeding, inflammatory response to heparin and protamine [2]

CPP, cerebral perfusion pressure; CBF, cerebral blood flow; CPB, cardiopulmonary bypass; BBB, blood-brain barrier; ICP, intracranial pressure; DHCA, deep hypothermic circulatory arrest; ACT, activated clotting time; CHD, congenital heart disease.

**Table 1c:** Postoperative Risk Factors for Perioperative Cerebral Injuries and Poor Neurodevelopmental Outcomes

Risk Factor	Examples
Hypoxemia [6]	Intrapulmonary shunting, pulmonary dysfunction, and continued venous and arterial mixing
Hemodynamic instability [6]	Cardiac dysfunction, new shunt physiology, mechanical ventilation, pulmonary hypertension, fluid shifts, sedation
Anemia [82]	
Inflammation	Cerebral edema with risk of elevated ICP, reperfusion injury [11,87]
Elevated CVP decreases CPP and CBF	Obstructed bidirectional cavopulmonary anastomosis, SVCS [27], pulmonary hypertension, right ventricle dysfunction, high mechanical ventilation pressures
Elevated ICP decreases CPP	Post-bypass cerebral edema secondary to microemboli [1] or inflammation and reperfusion injury [11,87]
Hyperthermia	Neuronal damage [12], possibly due to unmet cerebral oxygen requirements
Single-ventricle heart disease	Increased risk of post-operative cerebral emboli [12]. Persistent hypoxemia [25] may be secondary to the CHD and shunt physiology or induced by clinicians to balance pulmonary and systemic circulatory blood flow. Iatrogenic hypocapnia through hyperventilation decreases pulmonary blood flow and increases systemic blood flow at the expense of cerebral vasoconstriction [7] with decreased cerebral oxygen delivery.

ICP, intracranial pressure; CVP, central venous pressure; CPP, cerebral perfusion pressure; CBF, cerebral blood flow; SVCS, superior vena cava syndrome; CHD, congenital heart disease.

**Table 2:** Advantages and Limitations of Commonly Used, Noninvasive Neuromonitoring Techniques during CPB

Technique	Advantages	Limitations
NIRS	Inexpensive; user friendly; indicates ongoing or impending cerebral ischemia through trends on a numeric scale; measuring oxygen consumption is one of the most reliable measures of brain metabolic activity [32]; detects differential perfusion pressure and oxygenation between cerebral hemispheres [47,50]; can continuously assess cerebral autoregulation through the COx.	May not detect ischemia to deep brain structures; measures oxygenation to only regional frontal cerebral cortex; wide inter-patient variability; reading may change depending on where the probe is positioned on the forehead; 75% of reading is from venous saturation.
EEG	Detects seizures that are not clinically apparent due to pharmacologic sedation and neuromuscular blockade; detects subclinical seizures; identifies burst suppression or electric silence during cooling [38].	Technically difficult to use and interpret; numerous electrodes.
BIS	Simplified, user-friendly processed EEG; indicates cerebral ischemia [19,58] and awareness under anesthesia [58,62,63] through trends on a numeric scale; identifies burst-suppression or electrical silence during cooling [38].	Not well tested in children younger than 2 years [38]; readings are not changed by benzodiazepines or opioids, which makes it difficult to assess the risk of awareness during rewarming if these medications are given [38].
TCD	Assesses cerebral autoregulation across changes in blood pressure and temperature [38]; can measure CBF once cerebral autoregulation is lost during hypothermic CPB [38,50,53]; identifies threshold flow rates for minimal cerebral perfusion during low-flow CPB or RLFP [38]; detects cerebral emboli [38].	Cannot measure CBF volume if cerebral vascular resistance and perfusion pressure are changing; technically difficult to measure a specific cerebral artery when pulsatile perfusion is lost during CPB; only measures regional CBF.

NIRS, near-infrared spectroscopy; COx, cerebral oximetry index; EEG, electroencephalogram; BIS, Bispectral Index; TCD, transcranial Doppler ultrasonography; CBF, cerebral blood flow; CPB, cardiopulmonary bypass; RLFP, retrograde low flow perfusion.

**Table 3:** Treatment Algorithm Based on Changes in Neuromonitoring Parameters for Congenital Heart Surgery

Neuromonitor	Normal	Abnormality	Intervention
Cerebral Oximetry (%)	70%–90%	Pre/Post/During CPB: >20% reduction from baseline	Pre/Post/During CPB—increase CO, increase flow, adjust cannulae, increase PaCO_2_, increase MAP, increase temp, increase Hg, cross-correlate with other neuromonitors
		During DHCA<30%	DHCA—cerebral reperfusion
BIS/EEG	>80/normal f/amp	Pre/Post/During CPB: >60/increased f/amp	Pre/Post CPB—increase anesthesia; if persists, consider seizure; cross-correlate with other neuromonitors
		<30/decreased f/amp (during rewarming)	During CPB—increase anesthesia on pump, lower temp
		During DHCA: Not 0/not isoelectric	DHCA—continue cooling before DHCA
TCD (mean CBFV)	Normal (see table 4) or baseline CBFV	Pre/Post/During CPB: >25% decrease from baseline	Pre/Post CPB—evaluate TCD transducer position, cross-correlate neuromonitors (i.e., check cerebral oximeter; if low, increase CO, MAP, PaCO_2_) During CPB—as above, increase pump flow, check/adjust cannulae
		>25% increase from baseline	Pre/Post/During CPB—increase anesthesia depth, decrease PaCO_2_, MAP
		HITS	Pre/Post CPB—possible emboli. De-air lines, evaluate cannulae, stop infusions, lower head (Trendelenburg), evaluate with TEE. During CPB—use above de-airing maneuvers; consider slow wean on CPB, check EKG.

CPB, cardiopulmonary bypass; DHCA, deep hypothermic circulatory arrest; CO, cardiac output; Hg, hemoglobin; PaCO2, arterial carbon dioxide content; MAP, mean arterial pressure; BIS, bispectral index; EEG, electroencephalography; f, frequency of electrical EEG waves; amp, amplitude of electrical EEG waves; TCD, transcranial Doppler; CBFV, cerebral blood flow velocity; HITS, high intensity transient signals; TEE, transesophageal echocardiography; ECG, electrocardiography. (Adapted from [38] and [112]).

**Table 4:** Normal Transcranial-Doppler Velocities for Infants, Children, and Adults

Age	Depth (mm)	Mean Velocity (cm/s)	Peak Systolic Velocity (cm/s)	End–diastolic Velocity (cm/s)
0–3 months	25	(24–42)±10	(46–75)±15	(12–24)±8
3–12 months	30	74±14	114±20	46±9
1–3 years	35–45	85±10	124±10	6±11
3–6 years	40–45	94±10	147±17	65±9
6–10 years	45–50	97±9	143±13	72±9
10–18 years	45–50	81±11	129±17	60±8
18–40 years	45–50	81±20	120±28	55±13
41–60 years	45–50	73±19	109±22	49±13

Normal transcranial-Doppler velocities in the middle cerebral artery monitored through the temporal window in awake subjects without congenital heart disease (mean ± SD). (Adapted from [65,66,67].)
